# Feasibility of laparoscopic radical prostatectomy initiation by a surgeon with limited open surgery experience: A single-surgeon series with prior laparoscopic background

**DOI:** 10.1097/MD.0000000000050575

**Published:** 2026-09-04

**Authors:** Yusuf Senoglu, Emre Ediz, Ismail Eyup Dilek, Necati Ekici, Dursun Baba

**Affiliations:** a Department of Urology, School of Medicine, Marmara University, Istanbul, Turkey; b Department of Urology, Düzce University Faculty of Medicine, Düzce, Turkey.

**Keywords:** experience, laparoscopic prostatectomy, laparoscopic vs open surgery, radical prostatectomy

## Abstract

Due to its greater accessibility and cost-effectiveness, laparoscopic radical prostatectomy (LRP) is widely preferred worldwide. The aim of this study was to evaluate the outcomes of laparoscopy performed with prior limited open surgical experience. It retrospectively compares the initial cases of open radical prostatectomy (ORP) and LRP conducted by a single surgeon. A total of 54 patients were included in the study. From June 2019 to March 2021, 27 patients diagnosed with clinically localized prostate cancer underwent ORP, while from March 2021 to March 2022, 27 patients underwent LRP. Both groups were analyzed retrospectively regarding operative time, blood loss, nerve-sparing surgery, lymph node dissection, postoperative complications, and functional outcomes. There was no significant difference in mean operative time between the groups (266.8 vs 251.8 minutes). The mean blood loss was significantly lower in the LRP group (218.1 vs 987.7 mL, *P* < .001); 12 patients required blood transfusion in the ORP group. The hospital stay was significantly shorter in the LRP group (5.1 vs 6.2 days, *P* = .02). Positive surgical margin rates were 11% in the LRP and 4% in the ORP group (*P* = .30). Continence (16 vs 19, *P* = .40) and erectile dysfunction rates (70% vs 74%, *P* = .84) in LRP and ORP groups were similar. At 1-year follow-up, serum prostate-specific antigen levels were found to be <0.01 ng/mL in 24 patients in the LRP and 26 patients in the ORP group. This study suggests that LRP can be initiated by a surgeon with prior laparoscopic experience in other surgeries and limited ORP experience, provided an experienced surgical team and a structured training background are available, with acceptable perioperative, functional, and short-term oncological outcomes.

## 1. Introduction

Radical prostatectomy is a curative surgical treatment for localized prostate cancer that can be performed using open, laparoscopic, and robot-assisted laparoscopic techniques.^[1]^ It has long been known that the most important factor for achieving better functional outcomes, such as the preservation of postoperative continence and erectile function, is the surgeon’s experience.^[2]^ Therefore, given the complexity of the surgery, a surgeon must first gain sufficient experience with the procedure to perform it optimally.

Due to the long and challenging learning process, particularly in inexperienced clinics, there is no standardized method or established learning curve for radical prostatectomy.^[3]^ Numerous studies have shown that high-volume surgeons achieve better outcomes regarding complication rates, hospital stay durations, and postoperative functional recovery.^[4,5]^ The learning curve for radical prostatectomy in terms of surgical margins has been defined as 200 to 250 cases; however, the reduction rate in positive surgical margins (PSMs) has been reported to occur more rapidly with open radical prostatectomy (ORP).^[6]^ It has also been shown that the learning process takes longer with the laparoscopic approach.^[7]^ In a systematic review, the learning curve for operative time ranged from 10 to 250 cases for robot-assisted radical prostatectomy (RARP) and 40 to 250 cases for laparoscopic radical prostatectomy (LRP).^[8]^ Although RARP has a shorter learning curve than the standard technique, due to its greater accessibility and cost-effectiveness, standard LRP continues to be practiced in many countries.

In our study, we aimed to retrospectively compare the efficacy and safety of the first series of ORP cases performed by a single surgeon with the first series of LRP cases performed by the same surgeon. We evaluated oncological and functional outcomes at 1 year.

## 2. Materials and methods

This is a retrospective, single-center, observational study comparing the initial outcomes of ORP and LRP performed consecutively by a single surgeon. The local institutional ethics committee (February 19, 2024; Decision Number: 2024/12) approved the study protocol, and informed consent forms for participation in the study were obtained from all patients.

The study population was divided into 2 groups based on the surgical approach used: the ORP group, which included the first 27 patients operated on with an open approach between June 2019 and March 2021, and the LRP group, which included 27 consecutive patients operated on with a laparoscopic approach by the same surgeon between March 2021 and March 2022. Inclusion criteria were: biopsy-proven localized prostate cancer, eligibility for radical prostatectomy, and no prior pelvic radiotherapy or surgery. Patients with evidence of metastatic disease, prior hormonal therapy, or loss of follow-up within 12 months of surgery were excluded from the study. Patients were selected consecutively based on the order of presentation and surgical planning, without any selection based on tumor characteristics, age, comorbidities, or anticipated surgical difficulty. The preoperative and postoperative parameters of the 2 groups were evaluated retrospectively.

The surgeon, who had completed 5 years of urology training, accompanied a mentor in uro-oncological surgeries for approximately 2 years. During this period, the surgeon was the first assistant in 20 ORP and observed more than 50 LRP cases live in the operating room. Before performing LRP, the surgeon had around 2 years of laparoscopic experience in various urological procedures.

The surgeon independently performed his first ORP in June 2019 and completed 27 cases, approximately twice the time compared to the other group. This is because clinics were closed occasionally, and elective surgeries could not be performed due to the COVID-19 pandemic.

All patients underwent ORP using a midline incision below the umbilicus, following the technique described by Walsh.^[9]^ Six separate sutures with 3/0 atraumatic polyglactin were used for urethrovesical anastomosis.

LRP was conducted via the transperitoneal approach in a 30° Trendelenburg position with an insufflation pressure of 12 mm Hg. A 10-mm trocar placed superior to the umbilicus was used for the telescope. Subsequently, two 10-mm and two 5-mm trocars were inserted under endoscopic guidance. Urethrovesical anastomosis was performed continuously using a double-needle 3/0 absorbable barbed suture.

Patients in both groups were stratified according to the D’Amico risk classification. Pelvic lymph node dissection (PLND) was routinely performed in patients with high-risk disease. In patients with intermediate-risk disease, the decision to perform PLND was individualized based on preoperative clinical findings and the surgeon’s intraoperative assessment. During the initial evaluation, prostate volume was measured using prostate magnetic resonance imaging. Based on preoperative and intraoperative assessments, the neurovascular bundles were preserved in suitable patients. A 20 Fr indwelling urethral catheter was removed on the 14th postoperative day. All patients were advised to perform Kegel exercises before discharge and during postoperative follow-ups.

Intraoperative and early postoperative complications (within 28 days) were classified using the modified Clavien system. Anesthesiologists calculated the estimated blood loss by considering the volume collected in vacuum canisters, with the addition of the sponge count for ORP cases.

The evaluation criteria included preoperative data (patient age, preoperative prostate-specific antigen [PSA], prostate volume, biopsy International Society of Urological Pathology grade, and clinical stage), perioperative parameters (operative time, complications, blood loss, number of patients undergoing lymph node dissection, and nerve-sparing surgery), oncological outcomes (PSM rates, 12-month biochemical recurrence [BCR]), and functional outcomes (urinary continence and sexual function adequacy at 12 months).

Urinary continence and erectile function were assessed using the same predefined questions for all patients at routine postoperative follow-up visits scheduled at 3, 6, and 12 months. When an outpatient visit was not feasible, the same assessment was performed by telephone interview. Functional outcomes reported in the present study were based on the 12-month evaluation. Urinary continence was defined as complete daytime dryness or the use of no more than 1 safety pad per day. Erectile function was defined as the ability to achieve an erection sufficient for vaginal intercourse, with or without phosphodiesterase type 5 inhibitor therapy, according to patient self-report. BCR was defined as having 2 consecutive PSA measurements of ≥0.2 ng/mL.

IBM Statistical Package for the Social Sciences v27.0 software was used for statistical analysis, providing mean and standard deviation values for normally distributed data. The comparison of qualitative variables was performed using chi-square tests. Continuous variables were analyzed based on their distribution for comparisons between groups: parametric data were evaluated using the independent *t* test, while nonparametric data were assessed using the Mann–Whitney *U* test. The results were considered statistically significant at a 95% confidence interval with a significance level of *P* < .05.

## 3. Results

The demographic and preoperative parameters of patients who underwent radical prostatectomy are summarized in Table 1. Both groups were similar regarding patient age, PSA levels, prostate volume, International Society of Urological Pathology grade, and clinical stage.

**Table 1:** Preoperative characteristics of the patients.

	ORP	LRP	*P* value
Number of patients	27	27	>.99
Mean age ± SD	66.1 (±6.5)	67.1 (±7.4)	.60
Mean serum PSA (ng/mL) (min–max)	8.15 (0.4–52)	8.19 (3.06–24.5)	.98
Mean prostate volume (cc) (min–max)	49.3 (16–147)	50.1 (11–99)	.91
ISUP grade, n (%)			>.99
Grade 1	15 (56%)	14 (52%)	
Grade 2	9 (33%)	10 (37%)	
Grade 3	2 (7%)	3 (11%)	
Grade 4	1 (4%)	0 (0%)	
Clinical stage, n (%)			.79
cT1	12 (44%)	13 (48%)	
cT2	15 (56%)	14 (52%)	

*P* values calculated using independent *t* tests for continuous variables and chi-square tests for categorical variables.

ISUP = International Society of Urological Pathology, LRP = Laparoscopic Radical Prostatectomy group, min–max = minimum and maximum values, ORP = Open Radical Prostatectomy group, PSA = prostate-specific antigen, SD = standard deviation.

In the first 27 LRP cases, none required conversion to open surgery. Operative and perioperative findings are summarized in Table 2. The 2 groups had no statistically significant difference in the mean operative times. The estimated mean blood loss was 987.7 mL (70–5000 mL) in the ORP group and 218.1 mL (25–750 mL) in the LRP group (*P* < .001). While no blood transfusions were required in the LRP group, 12 patients in the ORP group received intraoperative blood transfusions.

**Table 2:** Operative data of the patients.

	ORP	LRP	Absolute differences	*P* value
Mean operative time (min) ± SD	266.8 (±50.6)	251.8 (±54.8)	15	.30
Mean blood loss (mL) (min–max)	987.7 (70–5000)	218.8 (25–750)	768.9	<.001
Number of patients receiving blood transfusion, n (%)	12 (44%)	0 (0%)	12 (44%)	<.001
Lymph node dissection, n (%)	15 (55%)	5 (19%)	10 (36%)	.004
Early postoperative complications, n (%)	1 (4%)	3 (11%)	−2 (−7%)	.30
Nerve-sparing surgery, n (%)	23 (85%)	24 (89%)	−1 (−4%)	.69
ISUP grade, n (%)				.12
Grade 1	8 (30%)	3 (11%)	5 (19%)	–
Grade 2	11 (40%)	13 (47%)	−2 (−7%)	–
Grade 3	1 (4%)	1 (4%)	0	–
Grade 4	6 (22%)	5 (19%)	1 (83%)	–
Grade 5	1 (4%)	5 (19%)	−4 (−15%)	–
Positive surgical margin, n (%)	1 (4%)	3 (11%)	−2 (−7%)	.30

*P* values calculated using independent *t* tests for continuous variables and chi-square tests for categorical variables.

ISUP = International Society of Urological Pathology, LRP = Laparoscopic Radical Prostatectomy group, min–max = minimum and maximum values, ORP = Open Radical Prostatectomy group, SD = standard deviation.

PSMs were detected in 11% (n = 3) of patients in the LRP group and 4% (n = 1) in the ORP group (*P* = .30). The rates of nerve-sparing techniques were similar in both groups. Obturator lymph node dissection was performed in 15 cases in the ORP group and 5 patients in the LRP group. Metastatic lymph nodes were detected in 2 patients in each group who underwent lymph node dissection.

Regarding early postoperative complications, 1 patient in the ORP group experienced lumbar pain, while 3 patients in the LRP group had Clavien-Dindo Grade I complications, such as skin ecchymosis and wound infections. No Clavien-Dindo Grade II or higher complications were observed in either group during the 30-day postoperative period.

The postoperative follow-up data for both groups are summarized in Table 3. A significant difference favoring the LRP group was observed concerning the average hospital stay. The mean length of hospital stay was 5.1 days (4–8) for the LRP group and 6.2 days (4–13) for the ORP group (*P* = .02).

**Table 3:** Postoperative data of the patients.

	ORP	LRP	Absolute differences	*P* value
Mean hospital stay (d) (min–max)	6.2 (4–13)	5.1 (4–8)	1.1	.02
Urinary continence, n (%)				.40
Incontinent	8 (30%)	11 (41%)	−3 (−11%)	
Continent	19 (70%)	16 (59%)	3 (11%)	
Erectile dysfunction, n (%)				.84
Present	20 (74%)	19 (70%)	1 (4%)	
Absent	7 (26%)	8 (30%)	−1 (−4%)	

*P* values calculated using independent *t* tests for continuous variables and chi-square tests for categorical variables.

LRP = Laparoscopic Radical Prostatectomy group, min–max = minimum and maximum values, ORP = Open Radical Prostatectomy group.

All patients underwent follow-up examinations every 3 months during the first year after radical prostatectomy. At the 1-year follow-up, when evaluating PSA levels and functional outcomes, continence was maintained in 16 patients (59%) in the LRP group and 19 patients (70%) in the ORP group (*P* = .40). PSA levels were measured as <0.01 ng/mL in 24 patients from the LRP group and 26 from the ORP group.

Erectile dysfunction was observed in 74% of patients in the ORP group and 70% of patients in the LRP group (*P* = .84).

## 4. Discussion

Surgical techniques for radical prostatectomy have evolved due to technological advancements and are performed in various forms, including perineal, retropubic, laparoscopic (transperitoneal and extraperitoneal), and robot-assisted laparoscopic approaches.

It remains difficult to determine the exact number of cases required to achieve proficiency in radical prostatectomy, as the learning curve is influenced by multiple surgeon-related, patient-related, and institution-related factors.^[10]^ For open and laparoscopic approaches in radical prostatectomy, the generally reported number of cases required to reach proficiency is around 200 to 250.^[6,8]^ A literature review reveals that studies on learning curves have used various endpoints, such as operative time, BCR, PSMs, and functional outcomes. It is possible to observe different numbers of cases required to reach a steady plateau for each of these parameters. In most studies, conclusions have been drawn by evaluating the surgical outcomes of multiple surgeons.^[6,11]^ The present study aimed to explore whether LRP could be initiated with acceptable perioperative, functional, and short-term oncological outcomes by a single surgeon with limited independent ORP experience but prior exposure to ORP as an assistant and previous laparoscopic experience in other urological procedures.

When considered together with broader evidence from large cohorts and systematic reviews, Sivaraman et al performed a cumulative pooled analysis of LRP outcomes and showed that meaningful competence may begin after approximately 40 to 60 cases, particularly in high-volume centers with experienced teams.^[5]^ Similarly, a comprehensive review by Chahal et al highlighted that the learning curve for laparoscopic procedures is significantly influenced by institutional resources and prior laparoscopic exposure, particularly in settings without robotic assistance.^[8]^ These findings highlight the importance of structured support systems and training infrastructure, particularly for surgeons working in resource-limited settings.

Robotic surgery, which has a significantly shorter learning curve than other methods, is becoming increasingly common worldwide. However, despite its many advantages, its high cost remains a significant obstacle to its widespread use.^[12]^ Therefore, ORP and LRP remain widely used as current surgical methods.

In ORP, it is anatomically and technically difficult to follow the surgical steps and see the technique. Usually, it can be learned with the primary surgeon’s direct assistance. On the other hand, the surgical area can be seen more clearly with laparoscopy. Although our study did not include a formal assessment of the learning curve, previous literature suggests that proficiency in LRP typically requires a longer learning process compared to ORP.^[11]^ During the learning process of LRP, evaluating intraoperative video recordings alongside pathological findings has been reported to be effective in enhancing surgical techniques, particularly in understanding how PSMs occur.^[13,14]^ Although the contribution of observational experience and supplementary video-based learning cannot be quantified in the present study, previous literature suggests that structured exposure, technical standardization, and progressive experience may facilitate the transition to complex minimally invasive procedures.^[15]^

For LRP, the surgical team’s experience supporting the surgeon in the early stages of their career is highly important. Unfamiliarity with surgical instruments, challenges related to surgical assistance, and patient positioning can affect not only the surgeon but also the primary assistant, nurses, anesthesiologists, and the entire operating room team. Therefore, working with a well-trained and motivated team is crucial for the success of a surgeon performing a procedure for the first time. In our study, no significant technical difficulties or severe complications were encountered since the surgical team, apart from the surgeon, had experienced both techniques before. In cases where both the surgeon and the operating room team lack experience, the learning process would be considerably more complex.^[16]^

Our study does not determine which technique is superior or should be preferred when starting radical prostatectomy. Instead, it evaluates what inexperienced surgeons may encounter in both the early and late periods of their initial cases using either technique. To understand this, it is essential to assess operative parameters, complications, erectile function, and continence as an integrated whole.

Many studies have found that operative times for initial cases are similar across open, laparoscopic, and robot-assisted surgeries. Previous research has shown that operative time generally decreases with increasing surgical experience during the learning curve for minimally invasive radical prostatectomy.^[17,18]^ In our study, no differences in operative times were observed between the 2 groups.

Despite being initial laparoscopic cases, blood loss was significantly lower, and no transfusions were required in our study. The primary causes of blood loss are bleeding from the dorsal vein complex and venous sinuses. According to current literature, intraoperative blood loss is significantly higher in ORP.^[19]^ The improved visualization of anatomical structures in laparoscopic surgery and the tamponade effect of pneumoperitoneum pressure on veins and venous sinuses reduce bleeding.^[20]^

The significantly lower rate of PLND in the LRP group deserves consideration. Although differences in preoperative risk distribution may have contributed to this imbalance, they are unlikely to fully explain the observed discrepancy. It probably also reflects technical and surgeon-related factors associated with the early laparoscopic learning phase. Laparoscopic PLND is technically more demanding than the open approach because it requires advanced dissection skills in a confined operative field despite excellent visualization. This observation may reflect a more selective approach during the early laparoscopic learning phase. Consequently, the lower PLND rate in the LRP group should be interpreted as a multifactorial finding rather than a difference attributable solely to patient risk stratification. This imbalance should also be considered when interpreting the oncological findings, as the lower frequency of PLND in the LRP group may have reduced the accuracy of pathological nodal staging and limited direct comparison of short-term oncological outcomes between the 2 groups.

Evaluating functional outcomes, particularly continence and potency, is mostly patient-dependent. In a study comparing ORP and RARP in the early learning period of a single surgeon, Philippou et al found that continence and potency rates were similar at 3 months postoperatively.^[17]^ Menon et al reported that, due to reduced bleeding and more precise visualization, critical structures for continence are better preserved during apical dissection with the robot-assisted laparoscopic technique.^[21]^ In our study, the continence rates were higher in the ORP group, although this was not statistically significant (70% vs 59%). This finding may support evidence that the learning curve for laparoscopy is slower during the initial phase.

It is well known that the likelihood of recovering erectile function after radical prostatectomy increases as the surgeon’s experience with the same technique improves.^[22]^ Hara et al compared the quality of life after ORP and LRP and found that potency outcomes after radical prostatectomy were nearly the same for both techniques.^[23]^ Our study observed potency at similar rates in the ORP and LRP groups.

In their retrospective analysis of 7765 patients, Vickers et al reported that the overall PSM rate was 27%, even in robot-assisted laparoscopic surgery.^[24]^ The PSM rate observed in the LRP group falls within the range reported in previous learning curve series; however, given the small sample size and limited follow-up period, this finding should be interpreted cautiously and should not be considered as evidence of oncological equivalence between the 2 approaches.

Previous ORP experience or the surgeon’s background does not seem to reduce PSM rates, suggesting that these outcomes are primarily a function of laparoscopic experience.^[6]^ It has been reported that the absolute risk of BCR increased from 7.8% to 20.1% in surgeons who had performed approximately 100 ORPs before their first laparoscopic experience.^[11]^ Although this study provides important information on early surgical outcomes in converting from ORP to LRP, the limited sample size of 27 patients per group reduces its statistical power to detect small to moderate differences in key clinical outcomes such as urinary continence and PSMs. Therefore, the findings should be considered exploratory rather than definitive.

In our study, no significant difference was observed between the 2 groups regarding complications. In addition, no anastomotic strictures were observed in either group. However, studies have suggested that laparoscopic surgery may be superior due to better visualization, improved preservation of the urethra, and the creation of a more watertight anastomosis using continuous sutures.^[25]^ In addition, recording surgeries and video guidance afterward effectively reduce adverse events during complex laparoscopic procedures.^[26,27]^ Despite the lack of experience and the fact that both techniques were performed for the first time, no major complications were observed in our study. The absence of major complications may be related to the surgeon’s previous laparoscopic experience and the support of an experienced operating room team; however, the retrospective design and small sample size preclude any definitive conclusion regarding the independent effect of these factors.

The future of surgical management in prostate cancer will likely continue to evolve along with technological advancements and shifting training paradigms. While laparoscopic and robotic techniques are gaining wider adoption, ORP may still play a vital role in resource-limited settings where robotic platforms remain unavailable. Therefore, structured laparoscopic training can serve as a bridge in centers transitioning from ORP to minimally invasive approaches.

The study’s retrospective design may be considered a limiting factor for patient selection. However, patient selection for both groups was based on the same inclusion criteria, and thus, selection bias is thought to be minimized. One notable limitation of this study is its small sample size and single-surgeon, single-center design, which may reduce the generalizability of the findings. The results should therefore be interpreted with caution and considered exploratory. Although the number of cases is small, our study reflects the situation of a surgeon switching to LRP after limited ORP experience.

Another important limitation is that functional outcomes were assessed using patient self-report rather than validated patient-reported outcome measures. Although the same predefined assessment was used consistently throughout follow-up, validated instruments such as the International Index of Erectile Function, the Expanded Prostate Cancer Index Composite, or validated continence questionnaires may provide more objective and reproducible assessments of postoperative functional recovery. The use of such standardized instruments would facilitate comparisons with other published series and increase confidence in the reported functional outcomes.

In the future, a prospective study with a long follow-up period, designed so that the same surgeon performs 1 open and 1 laparoscopic method sequentially, may provide more objective data and shed light on the literature.

## 5. Conclusion

LRP remains widely performed globally, especially in centers where cost-effectiveness is a priority. This study presents a unique series comparing the initial ORP and LRP experiences of a single surgeon. The findings show that surgeons with prior laparoscopic experience in other urological procedures and limited ORP experience can initiate LRP with acceptable perioperative, functional, and short-term oncological outcomes, provided that adequate laparoscopic experience, structured training, and an experienced surgical team are available. The notably lower blood loss observed in the laparoscopic group may provide a significant advantage for initial cases during the perioperative period. The small sample size, single-surgeon/single-center design, and a follow-up period of only 1 year make it challenging to detect more subtle but clinically meaningful differences between the techniques and limit the generalizability of the results. Multicenter, long-term prospective studies with larger sample sizes are necessary to strengthen the validity of these findings and better evaluate long-term outcomes, including BCR, continence, and erectile function after LRP.

## Acknowledgments

The author sincerely thanks Prof Dr Ali Tekin, the mentor who provided surgical experience and guidance for the initial laparoscopic and open procedures.

## Author contributions

**Conceptualization:** Yusuf Senoglu.

**Data curation:** Yusuf Senoglu, Emre Ediz, Necati Ekici.

**Formal analysis:** Yusuf Senoglu, Ismail Eyup Dilek.

**Methodology:** Yusuf Senoglu, Emre Ediz.

**Supervision:** Ismail Eyup Dilek, Dursun Baba.

**Visualization:** Ismail Eyup Dilek.

**Writing – original draft:** Yusuf Senoglu, Necati Ekici, Dursun Baba.

**Writing – review & editing:** Yusuf Senoglu, Dursun Baba.
